# Genome Sequence of the Sponge-Associated *Ruegeria halocynthiae* Strain MOLA R1/13b, a Marine *Roseobacter* with Two Quorum-Sensing-Based Communication Systems

**DOI:** 10.1128/genomeA.00998-14

**Published:** 2014-10-09

**Authors:** Margot Doberva, Sophie Sanchez-Ferandin, Yoan Ferandin, Laurent Intertaglia, Julie Croué, Marcelino Suzuki, Philippe Lebaron, Raphaël Lami

**Affiliations:** a Sorbonne Universités, UPMC Université Paris 06, USR 3579, LBBM, Observatoire Océanologique, Banyuls-sur-Mer, France; b CNRS, USR 3579, LBBM, Observatoire Océanologique, Banyuls-sur-Mer, France; c Sorbonne Universités, UPMC Université Paris 06, UMR 7232, BIOM, Observatoire Océanologique, Banyuls-sur-Mer, France; d CNRS, UMR 7232, BIOM, Observatoire Océanologique, Banyuls-sur-Mer, France; e Sorbonne Universités, UPMC Université Paris 06, UMS 2348, Observatoire Océanologique, Banyuls-sur-Mer, France; f CNRS, UMS 2348, Observatoire Océanologique, Banyuls-sur-Mer, France

## Abstract

*Ruegeria halocynthiae* MOLA R1/13b is an alphaproteobacterium isolated from the Mediterranean sea sponge *Crambe crambe*. We report here the genome sequence and its annotation, revealing the presence of quorum-sensing genes. This is the first report of the full genome of a *Ruegeria halocynthiae* strain.

## GENOME ANNOUNCEMENT

*Ruegeria halocynthiae* MOLA R1/13b (MOLA culture collection no. WDCM911; see http://collection.obs-banyuls.fr/index.php) was isolated in 1:5 marine R2A agar (in 75% seawater) at 20°C, from the sponge *Crambe crambe* collected on 20 January 2010 from a 12-m depth at the Bay of Banyuls (48°28*'*823ʺN, 3°08*'*038ʺE, France). A major step in our isolation protocol is that sponge homogenates were acclimated for 24 h at 16°C with marine R2A added at a 1:200 final dilution, before strain isolation. Based on its 16S rRNA gene sequences, the strain is phylogenetically related to *Ruegeria halocynthiae* MA1-6 (99% sequence identity to 16SrRNA genes) and belongs to the *Rhodobacteraceae* (*Roseobacter* clade).

The strain was cultivated in 100 mL of marine broth 2216 medium (BD, Difco, Sparks, MD) at 25°C over 48 h. DNA was extracted using a cetyltrimethylammonium bromide (CTAB)-based method (1). Genome sequencing steps conducted by MrDNA platform (Texas) included fragmentation, ligation to sequencing adapters, and purification. Following the amplification and denaturation steps, libraries were sequenced in a pool. A total of 50 ng of DNA was used to prepare the library using the Nextera DNA sample preparation kit (Illumina). Library insert size was determined by an Experion automated electrophoresis station (Bio-Rad). The insert size of the libraries ranged from 300 to 850 bp (average, 500 bp). The library (12 pM) was loaded (in a pool) to a 600 Cycles v3 Reagent cartridge (Illumina), and the sequencing was performed on a Miseq sequencer (Illumina) and *de novo* assembled with NGEN v11 (DNASTAR, Inc). The genome was annotated using Prokka 1.7 (www.vicbioinformatics.com/software.prokka.shtml.

The sequence draft genome sequence of *Ruegeria halocynthiae* MOLA R1/13b is 4,320,292 bp, with a G+C content of 58.3%, including 4,205 coding sequences, 2 rRNAs, and 53 tRNAs. The genome annotation revealed the presence of genes involved in quorum sensing. Two autoinducer type 1 synthases homologous to *luxI* and 6 corresponding *luxR* receptors (2) are detected in the genome. The genome annotation also revealed 3 genes encoding RhtB/lysE-like proteins, known to be involved in long chain homoserinelactones transmembrane transport (3). This is the first report of quorum-sensing genes within the marine species *Ruegeria halocynthiae*. Thus, this draft genome reinforces previous observations suggesting that marine bacteria are able to communicate using quorum sensing in sponge microenvironments where these cells can be found at high concentrations (4, 5)

### Nucleotide sequence accession number.

The whole-genome shotgun project has been deposited at DDJB/EMBL/GenBank under the accession no. JQEZ00000000.
